# Efficacy of FDA-Approved Anti-Inflammatory Drugs Against Venezuelan Equine Encephalitis Virus Infection

**DOI:** 10.3390/v11121151

**Published:** 2019-12-12

**Authors:** Kenneth Risner, Aslaa Ahmed, Allison Bakovic, Stephanie Kortchak, Nishank Bhalla, Aarthi Narayanan

**Affiliations:** National Center for Biodefense and Infectious Diseases, George Mason University, Manassas, VA 20110, USA; krisner@masonlive.gmu.edu (K.R.); aahmed30@masonlive.gmu.edu (A.A.); abakovic@masonlive.gmu.edu (A.B.); skortcha@masonlive.gmu.edu (S.K.); nbhalla@gmu.edu (N.B.)

**Keywords:** Venezuelan equine encephalitis virus, inflammation, FDA approved anti-inflammatory compounds, antiviral efficacy, inhibition

## Abstract

Venezuelan equine encephalitis virus (VEEV) is a category B select agent pathogen that can be aerosolized. Infections in murine models and humans can advance to an encephalitic phenotype which may result in long-term neurological complications or death. No specific FDA-approved treatments or vaccines are available for the treatment or prevention of VEEV infection. Neurotropic viral infections have two damaging components: neuronal death caused by viral replication, and damage from the subsequent inflammatory response. Reducing the level of inflammation may lessen neurological tissue damage that often arises following VEEV infection. In this study, three commercially available anti-inflammatory drugs, Celecoxib, Rolipram, and Tofacitinib, were evaluated for antiviral activity in an astrocyte and a microglial model of VEEV infection. The inhibitors were tested against the vaccine strain VEEV TC-83, as well as the wild-type VEEV Trinidad donkey strain. Celecoxib, Tofacitinib, and Rolipram significantly decreased viral titers both after pre-treatment and post-treatment of infected cells. VEEV Trinidad Donkey (TrD) titers were reduced 6.45-fold in cells treated with 50 µM of Celecoxib, 2.45-fold when treated with 50 µM of Tofacitinib, and 1.81-fold when treated with 50 µM of Rolipram. Celecoxib was also shown to decrease inflammatory gene expression in the context of TC-83 infection. Overall, Celecoxib demonstrated potency as a countermeasure strategy that slowed VEEV infection and infection-induced inflammation in an in vitro model.

## 1. Introduction

Venezuelan equine encephalitis virus (VEEV) is a neurotropic arbovirus endemic to the Americas [1]. VEEV is a New World (NW) alphavirus of the family *Togaviridae* and is classified as a Group IV (+) ssRNA virus. VEEV is categorized as a select agent pathogen by the Centers for Disease Control and the United States Department of Agriculture due to its potential for being weaponized as a consequence of a very low infective dose and an ability to be aerosolized [2]. The aerosol infective dose of VEEV TrD in a BALB/c mouse model has been shown to be less than one plaque forming unit (PFU) [3]. Mosquito-transmitted infections can occur at doses as low as 10 to 1000 PFU [4]. VEEV was previously developed into a biological weapon during the Cold War [5]. Furthermore, as an RNA virus, VEEV has the potential to quickly generate novel mutations that may allow for epidemic spread by its mosquito vectors. Mutations in the E1 glycoprotein of Chikungunya virus (CHIKV), a related alphavirus, led to increased fitness in mosquitoes which caused a worldwide pandemic that still persists today [6]. More than 1.5 million people have been infected in countries bordering the Indian Ocean since the outbreak began [7]. Major epidemic outbreaks of VEEV in the 1960s resulted in the infection of as many as 200,000 humans in Columbia [2]. VEEV has also been detected as far north as Texas and Florida [1,2].

VEEV infection in humans presents with flu-like symptoms including high fever, headache, and malaise [8]. Progression to an encephalitic phenotype can occur in 10–15% of cases and may result in long-term neurological complications and damage. The mortality rate following VEEV infection in humans is ~1% [1,9]. Neurotropic viral infections cause nervous tissue damage principally through two mechanisms: direct neuronal cell death as a consequence of viral replication, and the associated tissue damage arising from the effects of high levels of inflammation [9,10,11,12]. VEEV infection of the central nervous system (CNS) following subcutaneous infection occurs due to viral spread from replication sites in the periphery; however, the mechanism for CNS entry has not been definitively established [13]. Recent studies have demonstrated that replication in mouse models occurs in the brain prior to blood-brain barrier disruption [9], with the resulting inflammation damaging the blood-brain barrier and leading to increased permeability which may lead to neuroinvasion and subsequently cause permanent neurological sequelae [9]. In addition, microglia, the resident macrophage cells of the CNS, react to the infection by releasing pro-inflammatory cytokines [14]. This suggests that therapies targeting modulation of the inflammatory response following VEEV infection may be a promising avenue of investigation when compared to those directly targeting viral replication.

Currently, the only treatment available following VEEV infection is supportive intensive care. There are no FDA-approved commercially available vaccines or antiviral drugs to treat exposure to VEEV. In this study, we attempt to identify the efficacy and antiviral potential of three FDA-approved anti-inflammatory drugs against VEEV. The tested inhibitors are FDA-approved anti-inflammatory drugs that reduce inflammation by targeting a variety of pathways. Celecoxib was FDA-approved in 1998 and originally marketed as anti-arthritis drug with the trade name of Celebrex [15]. Celecoxib is a cyclooxygenase-2 (COX-2) selective non-steroidal anti-inflammatory drug (NSAID). COX-2 is stimulated by inflammatory signals such as IL-1, IL-6, and IL-8, cytokines known to be induced by VEEV, and upregulates the production of potent pro-inflammatory cytokines, including prostaglandin E2 [14,16]. Inhibition of this pathway may slow viral dissemination and reduce tissue damage that results from inflammation.

Rolipram inhibits the phosphodiesterase-4 (PDE4) pathway [17]. It was discovered in the 1990s and initially investigated as an antidepressant [18]. PDE4 modulates the cyclic AMP pathway by degradation cAMP and has pro-inflammatory effects on a range of inflammatory cells including macrophages, T cells, and B cells [19,20]. Inhibition of the PDE4 pathway during VEEV infection may dampen the inflammatory response while still allowing adequate immune system activity against VEEV, thereby reducing tissue damage caused by excessive inflammation.

Tofacitinib is a Janus kinase (JAK) inhibitor, and specifically inhibits JAK1 and JAK3 activity. It was approved by the FDA in 2012 for the treatment of rheumatoid arthritis. Downstream signaling by the JAK-STAT signaling pathway mediates the upregulation of many pro-inflammatory cytokines, including IFNγ, IL-2, IL-4, and IL-10 [21], and inhibiting this pathway may reduce inflammation-mediated host tissue damage at sites of infection.

In this study, we tested the toxicity and efficacy of these drugs in the context of VEEV infection in the human microglial (HMC3s) and astrocyte (U87 MG) cell lines. In the nontoxic concentration of all inhibitors, we observed a significant decrease in viral titers following pre-treatment of cells with Celecoxib, Tofacitinib, or Rolipram with either the TC-83 or the virulent Trinidad Donkey (TrD) strains. In addition, post-exposure efficacy against the TC-83 strain was also observed following treatment of infected cells with all three tested drugs at 2, 4, and 6 hpi (hours post infection). Celecoxib exhibited the highest anti-VEEV activity among the tested inhibitors, with treatment inhibiting replication up to six hours after exposure. Furthermore, cytokine gene levels in infected cells were reduced after treatment with Celecoxib, most notably IL1A, IL17F and TNFα. Overall, Celecoxib demonstrated functionality and effectivity by delaying viral replication and infection-induced inflammation in VEEV-infected cells.

## 2. Material and Methods

### 2.1. Cell Lines, Viruses, and Reagents

Celecoxib (S1261), Rolipram (S2127), and Tofacitinib (S5001) were obtained from SelleckChem. The live-attenuated TC-83 vaccine strain of VEEV was acquired from BEI Resources. HMC3 human microglial cells (ATCC CRL-3304), U87 MG human brain astrocytoma cells (ATCC HTB-14), and VERO African green monkey kidney cells (ATCC CCL-81) were obtained from the American Type Culture Collection. U87 MG cells were maintained in Dulbecco’s Modified Eagle’s Medium (DMEM) (VWR Life Science VWRL0102-500) supplemented with 10% heat-inactivated fetal bovine serum (FBS) (Gibco 10,437,028), 1% Penicillin/Streptomycin (Gibco 15-140-122), and 1% L-Glutamine (VWR Life Science VWRL0131-0100) at 37 °C and 5% CO_2_. HMC3 cells were maintained in Eagle’s minimal essential medium (EMEM) (VWR Life Sciences 10128-214) supplemented with 10% FBS and 1% Penicillin/Streptomycin at 37 °C and 5% CO_2_. African green monkey kidney cells (VEROs) were maintained in DMEM supplemented with 5% heat-inactivated fetal bovine essence (FBE) (VWR Life Sciences 10803-034), 1% Penicillin/Streptomycin, and 1% L-Glutamine at 37 °C and 5% CO_2_. All reagents for cell maintenance were pre-warmed to 37 °C before use.

### 2.2. Viral Infections and Inhibitor Studies

In 96-well plates, HMC3 or U87 MG cells were seeded at a density of 3E4 or 1E4 cells/well, respectively. The inhibitors were resuspended in dimethyl sulfoxide (DMSO) prior to dilution to the indicated concentrations in culture media and cells were pre-treated before infection for 2 h. After 2 h, media was removed and saved, and is thereafter referred to as conditioned media. The virus was diluted in media and cells were then infected for 1 h at indicated multiplicities of infection at 37 °C, 5% CO_2_ to allow for the uptake of the virus. Viral inoculum was removed, cells were gently washed three times with Dulbecco’s phosphate-buffered saline (DPBS) without calcium and magnesium (Gibco 14190144), and conditioned media was placed onto the cells. Cells were incubated at 37 °C and supernatants were collected at indicated times post infection and stored at –80 °C.

### 2.3. Viral Plaque Assays

Plaque assays were performed using VERO cells grown to a concentration of 2E5 cells/well in a 12-well plate. Supernatants from infected cells were serially diluted in media and used to infect VERO cells for one hour. After infection, a 1:1 overlay consisting of EMEM (without phenol red, supplemented with 10% FBS, non-essential amino acids, 1 mM sodium pyruvate, 2 mM L-glutamine, 20 U/mL of penicillin, and 20 μg/mL of streptomycin) and 0.6% agarose was added to each well. Plates were incubated at 37 °C for 48 h. Cells were fixed with 10% formaldehyde for 1 h at room temperature. Formaldehyde was aspirated and the agarose overlay was removed. Cells were stained with crystal violet (1% CV *w*/*v* in a 20% ethanol solution). Viral titer (PFU/mL) of VEEV infection was determined by plaque count.

### 2.4. Cell Viability Assay

The viability of HMC3 and U87 MG cells treated with indicated inhibitors was determined using the CellTiterGlo assay (Promega). Inhibitors were resuspended in DMSO prior to dilution to tested concentrations in culture media. U87 MGs were seeded at 1E4 and HMC3s at 3E4. Inhibitors were overlaid on cells grown in a white-walled 96-well plate, which was incubated at 37 °C, 5% CO_2_ for 24 h. CellTiterGlo substrate was used according to the manufacturer’s instructions. Luminescence was determined using a DTX 880 multimode detector (Beckman Coulter) with an integration time of 100 ms/well. Cell viability was determined to be percent cell viability normalized to a DMSO-treated control.

### 2.5. Quantitative Real-Time Polymerase Chain Reaction

For QRT-PCR, samples were collected using Trysol reagent (ThermoFisher 15596026). RNA was extracted using the Zymo Research Direct-zol RNA Miniprep Kit (R2104) as per manufacturer’s instructions. Extracellular samples were collected from supernatants. Intracellular RNA was collected by direct cell lysis. The pre-cycling conditions were adapted from Verso 1-step RT-qPCR kit (ThermoFisher AB4101C) manufacturer’s instructions: 1 cycle at 50 °C for 20 min, 1 cycle at 95 °C for 15 min, 40 cycles at 95 °C for 15 s and at 51 °C for 1 min using a StepOnePlus™ Real Time PCR system (Applied Biosystems 4376600). VEEV TC-83 primers and probes targeted nucleotides 7931-8005 in VEEV capsid: forward primer (5’-TCTGACAAGACGTTCCCAATCA-3’) and reverse primer (5’-GAATAACTTCCCTCCGACCACA-3’) [22]. The probe utilized different tags (5ʹ6-FAM/TGTTGGAAG/ZEN/GGAAGATAAACGGCTACGC/3ʹIABkFQ) to improve sensitivity. Primers and probe were designed by and obtained from Integrated DNA Technologies (Skokie, IL). A standard curve was used to quantify levels of RNA based on threshold cycle (Ct) counts.

### 2.6. Statistical Analysis

Statistical analyses were performed using the software Prism 5 (Graph Pad). Data are presented as mean ± standard deviation (SD) after analysis with an unpaired, two-tailed t-test. Differences in statistical significance are indicated with asterisks: * *p* < 0.05; ** *p* < 0.01; *** *p* < 0.001; **** *p* < 0.0001. Number of replicates per experiment is indicated in each figure legend.

## 3. Results

### 3.1. Anti-Inflammatory Drug Toxicity in HMC3 Microglia and U87 MG Astrocyte Cells

**Figure 1:**
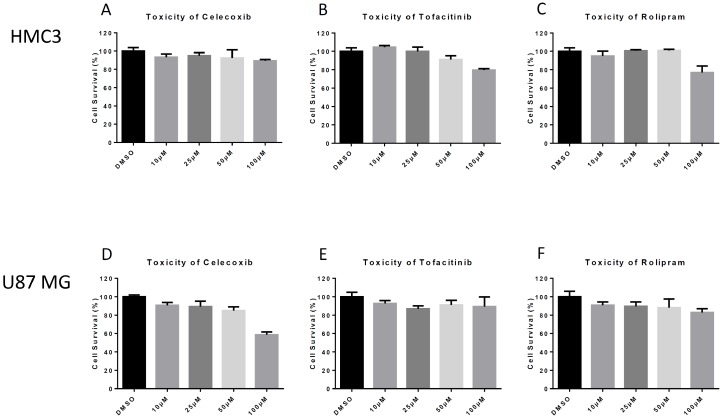
Drug Toxicity. Human microglial (HMC3) and astrocyte (U87 MG) cells were seeded at the appropriate density for the given cell line and incubated for 24 h. Cells were then overlaid with media containing indicated concentrations of each drug for 24 h. CellTiterGlo luminescence assay was used to determine cell viability. (**A**) Toxicity of celecoxib in HMC3s; (**B**) Toxicity of Tofacitinib in HMC3s; (**C**) Toxicity of Rolipram in HMC3s; (**D**) Toxicity of Celecoxib in U87 MGs; (**E**) Toxicity of Tofacitinib in U87 MGs; (**F**). Toxicity of Rolipram in U87 MGs. The quantitative data are depicted as the mean of three biologically independent experiments ± SD.

Microglia are macrophages of the central nervous system and play an important role in the induction of inflammation following VEEV infection [14]. Therefore, we used HMC3 human-derived microglia to evaluate the effects of various drugs on VEEV infection using a previously established model [23]. We first tested a range of concentrations for inhibitors in HMC3 cells to determine the optimum permissible drug concentration, defined by an appropriate balance between toxicity and viral replication inhibition. Cell survivability following inhibitor treatment was evaluated (Figure 1). At a concentration of 50 μM, Celecoxib, Rolipram, and Tofacitinib demonstrated low toxicity with cell survival at >90% (Figure 1A–C), whereas increasing toxicity was observed at a higher concentration (100 µM). Cell viability was standardized against the solvent DMSO used at a concentration of 0.1%. Therefore, a concentration of 50 μM was chosen for future experiments using HMC3 cells.

Astrocytes are a support cell of the central nervous system and help control permeability of the blood-brain barrier [24]. An astrocyte model of infection was also evaluated using U87 MG human astrocytes. Similar to the previous experiment, cell viability assays were conducted with Celecoxib, Tofacitinib, and Rolipram. Cell survivability decreased below 90% at concentrations of 25 μM and greater in U87 MGs (Figure 1D–F). Based on our data, a working concentration of 10 μM was selected for each drug for further analyses since cell survivability decreased upon treatment with higher drug concentrations (Figure 1D–F).

### 3.2. Anti-Inflammatory Drug Efficacy in VEEV Infection

**Figure 2:**
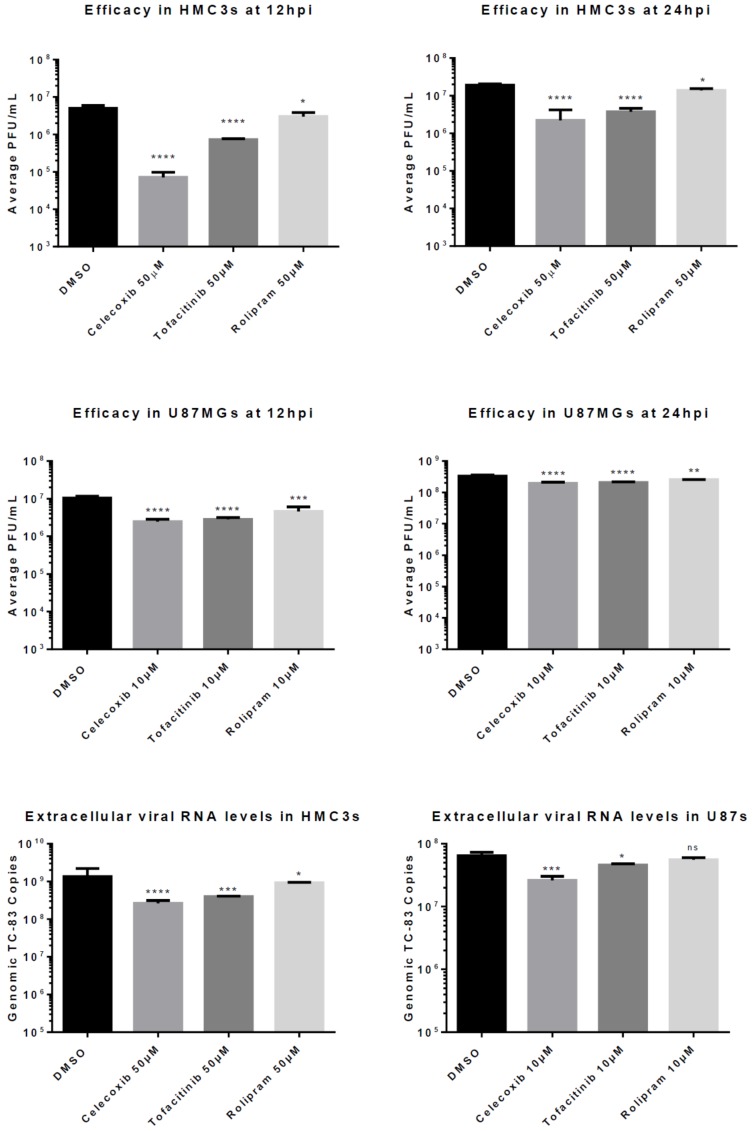
Drug Efficacy. HMC3s or U87 MGs were pre-treated with dimethyl sulfoxide (DMSO) or inhibitors at indicated concentrations for 2 h, then infected with TC-83 (MOI = 0.1). Supernatants were collected at 12 and 24 hpi to determine infectious viral titers. (**A**) Efficacy of 50 µM in HMC3s at 12hpi; (**B**) Efficacy of 50 µM in HMC3s at 24 hpi; (**C**) Efficacy of 10 µM in U87 MGs at 12 hpi; (**D**) Efficacy of 10 µM in U87 MGs at 24 hpi. Supernatants were collected at 24 hpi and extracellular viral load was quantified. Levels of viral RNA were measured by qRT-PCR; (**E**) Viral RNA levels in HMC3s treated with 50 µM of inhibitor at 24hpi; (**F**). Viral RNA levels in U87 MGs treated with 10 µM of inhibitor at 24hpi. The quantitative data are depicted as the mean of three biologically independent experiments ± SD. * *p* < 0.05; ** *p* < 0.01; *** *p* < 0.001; ****p < 0.0001.

The ability of the test inhibitors to reduce viral replication was evaluated in HMC3 and U87 MG cell lines. Cells were pre-treated with each inhibitor at a concentration of 50 μM in HMC3s and 10 μM in U87 MGs for 2 h and infected with VEEV TC-83 at an MOI (multiplicity of infection) of 0.1 (Figure 2). Supernatants were collected at indicated time points post infection and infectious viral titer was measured by plaque assay.

At 24 hpi in HMC3s, pre-treatment with all three tested anti-inflammatory drugs demonstrated a significant decrease in viral titer when compared to treatment with the negative control DMSO. Celecoxib and Tofacitinib pre-treatment decreased viral replication approximately 10-fold (*p* < 0.0001) when compared to 0.1% DMSO-treated cells. At 12 hpi, a 100-fold decrease (*p* < 0.0001) was observed in viral titers following Celecoxib pre-treatment when compared to the negative control (Figure 2A,B). In contrast, Tofacitinib pre-treatment reduced titers approximately 10-fold (*p* < 0.0001) whereas, pre-treatment with Rolipram reduced virus level 5-fold (*p* < 0.05) when compared to DMSO-treated cells.

Reduced viral titers following inhibitor pre-treatment were also observed in U87 MG cells. At 24 hpi, pre-treatment with all three inhibitors demonstrated a significant decrease in viral titers; however, the level of reductions in replication were lower when compared to treatment of HMC3 cells. Celecoxib and Tofacitinib pre-treatment significantly reduced VEEV replication (*p* < 0.0001) by 42% and 38%, respectively, when compared to replication in DMSO-treated cells (Figure 2D), whereas Rolipram reduced viral replication (*p* < 0.01) by 23%. At 12 hpi, celecoxib treatment demonstrated a 77% reduction (*p* < 0.0001), while Tofacitinib showed a 73% reduction (*p* < 0.0001) when compared to the negative control DMSO 0.1% (Figure 2C).

Additionally, RNA from U87 MG and HMC3 infected cells was collected at 24 hpi for analysis by qRT-PCR to determine reduction in levels of genomic viral RNA following inhibitor treatment. At 24 hpi, extracellular genomic copies in inhibitor-treated HMC3s demonstrated similar reduction to that observed by plaque assay (Figure 2E–F). In HMC3s Celecoxib, Tofacitinib, and Rolipram reduced titers approximately 5-fold (*p* < 0.0001), 3-fold (*p* < 0.001) and 1.5-fold (*p* < 0.05), respectively, when compared to DMSO-treated cells.

### 3.3. Efficacy of Inhibitors Against the Wild-Type VEEV TrD Strain

**Figure 3:**
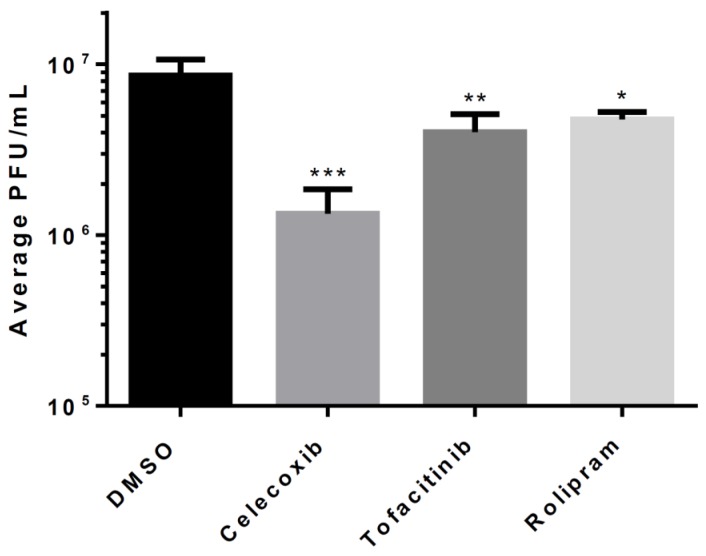
Efficacy against VEEV TrD. HMC3s were pre-treated with DMSO or inhibitors at indicated concentrations, infected with VEEV TrD (MOI = 0.1), and supernatants were collected at 16 hpi. Viral plaque assay was used to determine infectious viral titers. The quantitative data are depicted as the mean of three biologically independent experiments ± SD. * *p* < 0.05; ** *p* < 0.01; *** *p* < 0.001; **** *p* < 0.0001.

We next tested the efficacy of the inhibitors against the wild-type TrD strain of VEEV. HMC3s were pre-treated with 0.1% DMSO or one of the anti-inflammatory drugs at a concentration of 50 µM. Supernatants were collected at 18 hpi and infectious viral titers were determined by plaque assay (Figure 3). Similar to results obtained following TC-83 infection, Celecoxib demonstrated the greatest efficacy in controlling viral replication. Titers in treated cells were 6.45-fold lower when compared to the untreated cells (*p* < 0.0001), suggesting that the inhibitory effect of Celecoxib is effective against virulent strains of VEEV. Tofacitinib and Rolipram were less effective at controlling VEEV replication than Celecoxib. However, a statistically significant 2- to 3-fold reduction in replication was observed following Tofacitinib (*p* < 0.001) or Rolipram (*p* < 0.05) treatment when compared to DMSO-treated cells.

### 3.4. Post-Exposure Efficacy and Concentration Dependency of Celecoxib in HMC3s

**Figure 4:**
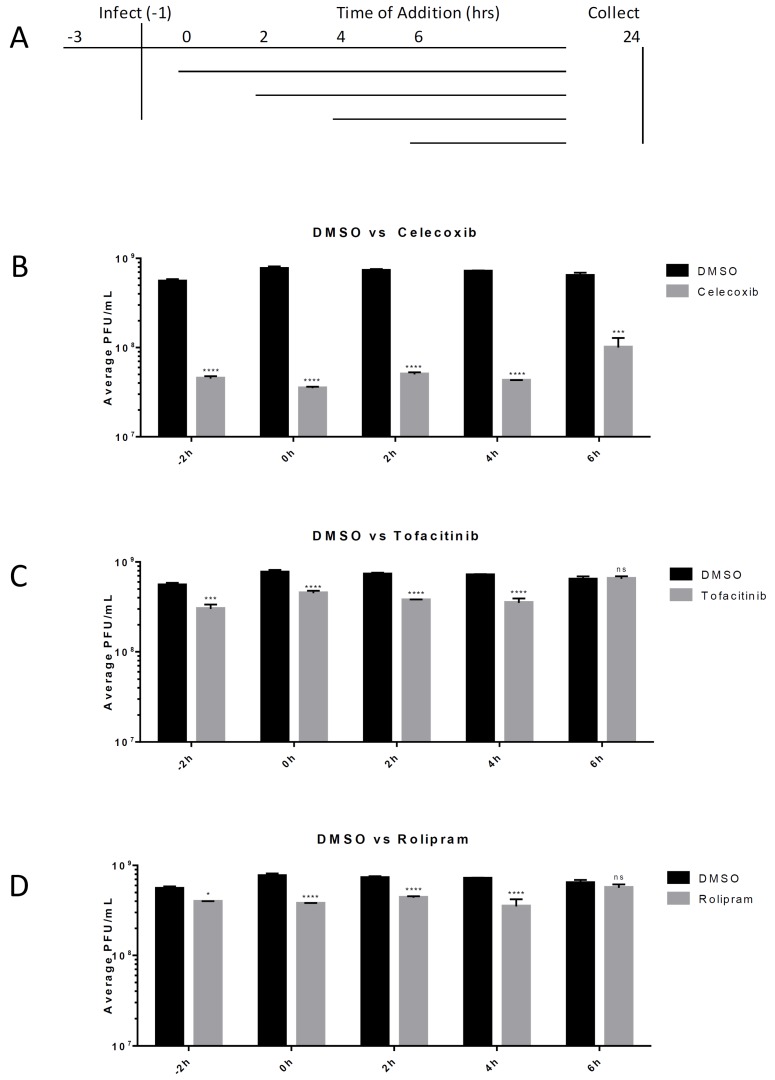
Post-exposure efficacy of inhibitors. HMC3s were infected with TC-83 (MOI = 0.1) for 1 hr. Viral inoculum was then removed, cells were washed with Dulbecco’s phosphate-buffered saline (DPBS) and fresh media was replaced. At 2 h intervals, DMSO or inhibitors at indicated concentrations were added to the media overlay. Supernatants were collected at 24 hpi and analyzed for infectious viral particles by viral plaque assay. (**A**) Schematic of the experimental design; (**B**) Efficacy of Celecoxib (50 µM); (**C**) Efficacy of Tofacitinib (50 µM); (**D**) Efficacy of Rolipram (50 µM). The quantitative data are depicted as the mean of three biologically independent experiments ± SD. * *p* < 0.05; ** *p* < 0.01; *** *p* < 0.001; ****p < 0.0001.

Post-exposure efficacy against VEEV infection was determined for the tested anti-inflammatory drugs. HMC3s were untreated or treated with 50 μM of the inhibitor Celecoxib, Rolipram, or Tofacitinib. Cells were treated with an inhibitor 2 h prior to infection, as well as 2 h, 4 h, or 6 h post-infection with VEEV TC-83 (MOI = 0.1). Supernatants were collected at 24 hpi and infectious viral titers were determined using a plaque assay. Celecoxib significantly reduced viral titers (*p* < 0.001) when infected cells were treated up to six hours after exposure to VEEV. At 2 and 4 hpi Tofacitinib and Rolipram treatment lowered titer approximately 2-fold (*p* < 0.0001), whereas Celecoxib treatment reduced levels more that 10-fold (*p* < 0.0001). For treatment at 6 hpi, Rolipram and Tofacitinib did not significantly reduce virus replication, whereas Celecoxib treatment demonstrated a greater than 6-fold reduction (*p* < 0.0001) in viral replication levels when compared to replication in DMSO-treated cells (Figure 4).

**Figure 5:**
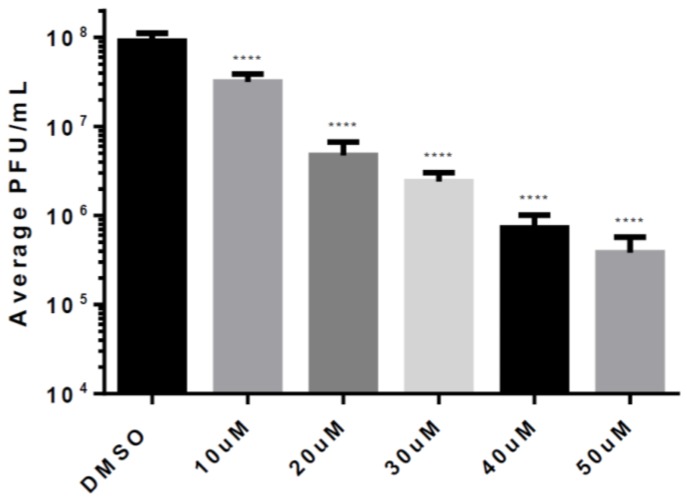
Dose response for Celecoxib. HMC3s were pre-treated with 50 µM Celecoxib for 2 h and infected with TC-83 (MOI = 0.1) for 1 hr. Viral inoculum was then removed, cells were washed with DPBS, and fresh media was replaced. Supernatants were collected at 12 hpi and analyzed for infectious viral particles by plaque assay. The quantitative data are depicted as the mean of three biologically independent experiments ± SD. * *p* < 0.05; ** *p* < 0.01; *** *p* < 0.001; ****p < 0.0001.

Based on the results of our previous data, we selected Celecoxib for more detailed studies, and evaluated the efficacy of Celecoxib treatment against VEEV infection at a range of concentrations. HMC3s were untreated or treated with increasing doses of Celecoxib that were below the toxic concentration. Treated cells were infected with VEEV TC-83 (MOI = 0.1), supernatants were collected at 12 hpi and replication was measured by plaque assay. Increasing concentrations of Celecoxib decreased viral titers in a linear manner (Figure 5). For every 10 µM increase in concentration, viral titers were reduced by 5-fold over DMSO-treated cells, suggesting that the efficacy of Celecoxib against VEEV is dose-dependent.

### 3.5. Celecoxib Treatment Reduces Levels of Pro-Inflammatory Cytokines

**Figure 6:**
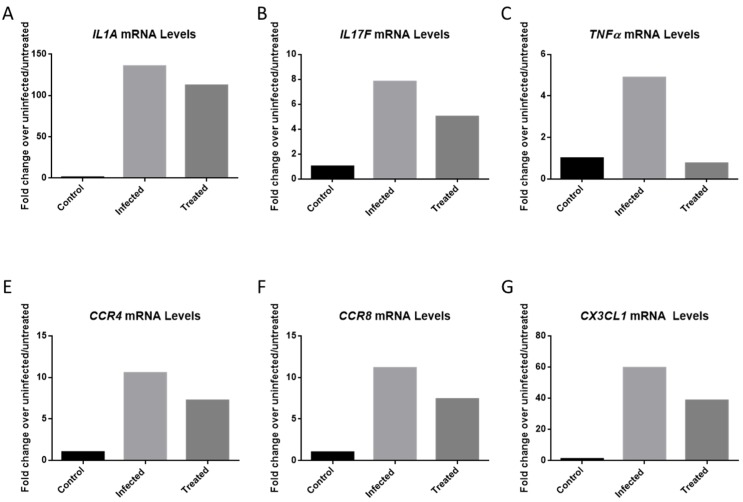
PCR Inflammatory Array. HMC3s were treated with DMSO or 50 µM Celecoxib for 2 hr prior to infection. Untreated, infected and treated, infected samples were infected with VEEV TC-83 (MOI = 0.1) and samples collected at 12 hpi. Qiagen’s RT2 Cytokine Array was used to quantify results. (**A**) mRNA levels of *IL1A*. (**B**) mRNA levels of *IL17F*. (**C**) mRNA levels of *TNFα*. (**D**) mRNA levels of *CCR4*. (**E**) mRNA levels of *CCR8.* (**F**) mRNA levels of *CX3CL1*.

VEEV infection results in the upregulation of many pro-inflammatory cytokines that can damage the blood-brain barrier which can lead to permanent damage to the central nervous system [9,10]. Elevated cytokine levels can result from microglia activation in response to a viral infection [25]. Cytokine levels from infected, inhibitor-treated and mock infected HMC3 samples were measured at 12 hpi. Cells were pre-treated for 2 h with 50 µM of Celecoxib, and subsequently infected with VEEV TC-83 (MOI = 0.1). The results demonstrate the upregulation of several cytokines following VEEV infection, which was reduced following treatment of infected cells with Celecoxib. The highest mRNA inductions were observed for IL1A, IL17F, CCR4, CCR8, CX3CL1 and TNFα (Figure 6).

VEEV infection raised mRNA copies of IL1A 135-fold when compared to mock infected cells, whereas levels of this mRNA in infected cells treated with Celecoxib were increased only 112-fold (Figure 6A). Similarly, tumor necrosis factor-α levels were increased 4.89-fold following viral infection, whereas treatment with Celecoxib brought TNFα levels back to basal levels (Figure 6C). CX3CL1 mRNA levels were reduced from 59.65- to 38.65-fold following treatment with Celecoxib. CCR4 was reduced from 10.54-fold to 7.23. CCR8 was reduced from 11.17 to 7.40. Overall, our data suggest that pre-treatment of cells with Celecoxib helped control the inflammation associated with VEEV infection of HMC3 cells.

### 3.6. Indirect Pro-Inflammatory Cytokines Increase in Viral Spread is Reduced by Celecoxib

**Figure 7:**
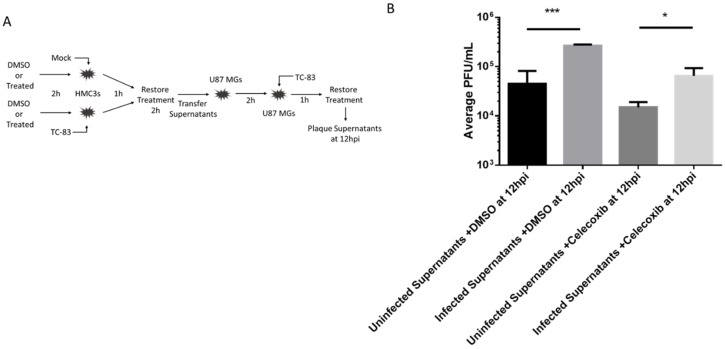
Viral inhibition following indirect inflammatory response. (**A**) Schematic for the experimental design. Supernatants were removed from DMSO or celecoxib treated, TC-83 inoculated HMC3 cells and overlaid onto naïve celecoxib treated or untreated U87 MG cells prior to inoculation of the astrocytes with TC-83. Viral inoculums were removed, and cells were incubated for 12 hpi. **(B**) Supernatants were removed and viral titers were determined by plaque assay. The quantitative data are depicted as the means of three biologically independent experiments ± SD. * *p* < 0.05; ** *p* < 0.01; *** *p* < 0.001; ****p < 0.0001.

It has been previously shown that HMC3 microglia release cytokines after being exposed to TC-83 and make the surrounding environment more susceptible to viral infection [14]. Here, we evaluated celecoxib’s ability to modulate such an inflammatory environment and the resulting infection. HMC3 were treated with DMSO or 10 µM of celecoxib for 2hrs. Treatment was removed and cells were infected with TC-83 (0.1 MOI) for one hour. Infection was removed and cells were washed with DPBS. Treatment was replaced for two hours. Supernatants were then removed and transferred to naïve U87 MGs that had been treated with celecoxib 10 µM or DMSO for two hours. After the U87 MGs incubated for two hours, supernatants were removed and cells were infected with TC-83 for 1 h. Infection was removed, cells were washed with DPBS, and fresh DMSO of celecoxib containing media was replaced. At 12 hpi, supernatants were removed and viral titers were determined by plaque assay (Figure 7). It was shown that U87 MGs produce a lot more virus after being exposed to primed HMC3 supernatants. Celecoxib was able to significantly reduce viral titers in U87 MGs after being primed with HMC3 supernatants. 

## 4. Discussion

VEEV is a category B select agent and an emerging infectious agent that is capable of causing systemic and neuronal disease upon exposure. There is currently no FDA-approved countermeasure strategy available to address VEEV infection and disease. VEEV-infection dependent neurological disease is characterized by a strong inflammatory component that may play a critical role in the onset of neurological manifestations. An ideal treatment strategy to deal with VEEV infections should therefore be capable of controlling not only the viral load, but also the inflammatory output and resulting damage to the tissue microenvironment.

With the intention of identifying countermeasure strategies, three anti-inflammatory drugs namely, Celecoxib, Tofacitinib, and Rolipram, were evaluated to determine their efficacy in controlling VEEV infection in an in vitro model of infection. We determined that Celecoxib was the most effective drug out of the three that were evaluated. Celecoxib inhibited VEEV TC-83 replication in multiple human cell lines, HMC3 microglia and U87 MG astrocytes, as well as the wild-type strain Trinidad Donkey in HMC3 cells. We observed lower levels of inhibition in U87 MG cells when compared to that observed in HMC3 cells. This may be due to the use of a lower dose of drug treatment in these cells due to toxicity observed at higher doses. Previous studies have shown that alphavirus infection activates microglia and astrocytes in vivo [26], and both cell types have been identified as mediators in the development of Venezuelan equine encephalitis through the production of pro-inflammatory cytokines following infection [14]. Astrocytes and microglia are both activated in vivo; however, it is unknown whether or not they are infected in vivo following infection. Both cell types have been shown to contribute to inflammation following viral infection by producing cytokines such as TNFα and inducible nitrous oxide synthase [7].

Previous in vivo studies using VEEV have shown that non-selective COX inhibitors can delay the onset of symptoms in a mouse model. In one study, mice were treated once per day with naproxen during the course of infection to evaluate the importance of inflammation in a VEEV infection model. Overall mortality in the study was not reduced for the treated cohort, but the onset of symptoms was delayed following naproxen treatment of infected mice [10]. Naproxen was not evaluated for any anti-viral properties and was chosen since it is a known anti-inflammatory drug. For these reasons, Celecoxib may be a viable candidate for an in vivo study testing its efficacy against VEEV.

We also evaluated the efficacy of Celecoxib against VEEV when administered to cells post-infection. Treatment reduced viral titers up to six hours after exposure to VEEV had occurred. This post-exposure efficacy of Celecoxib may be beneficial as a countermeasure strategy for treating VEEV infections and should be investigated in an animal model mimicking exposure to an intentional release or a lab-acquired infection.

Viral infections are known to induce pro-inflammatory cytokines [14]. Specifically, activation of microglia during a NW alphavirus infection causes activation of many inflammatory pathways, including IL-1, IL-6, and TNFα [12]. The inflammatory response can lead to an encephalitic phenotype that can cause permanent neurological damage or death [9,10]. The effect of Celecoxib treatment on these pathways was evaluated and Celecoxib treatment reduced the upregulation of several critical cytokines including IL-1α and TNFα following infection. Furthermore, cells primed with supernatants from previous VEEV exposure have also been shown to produce higher viral titers and cytokine concentrations [14]. Treatment with Celecoxib lowers both viral load and cytokine concentrations following indirect inflammatory exposure. Controlling the inflammatory environment during a neurotropic viral infection may be beneficial to preventing long-term neurological complications.

Overall, our data demonstrates that Celecoxib, an FDA-approved COX-2 inhibitor, is capable of slowing the replication of both TC-83 and a wild-type strain of VEEV in an in vitro model of infection. Furthermore, Celecoxib treatment reduces the upregulation of pro-inflammatory cytokines that can contribute to VEEV-mediated neurological damage following infection.
